# Rapid antibiotic susceptibility testing on blood cultures using MALDI-TOF MS

**DOI:** 10.1371/journal.pone.0205603

**Published:** 2018-10-11

**Authors:** Marlène Sauget, Xavier Bertrand, Didier Hocquet

**Affiliations:** 1 Hygiène Hospitalière, Centre Hospitalier Régional Universitaire, Besançon, France; 2 Centre de Ressources Biologiques Filière Microbiologique de Besançon, Centre Hospitalier Régional Universitaire, Besançon, France; 3 UMR CNRS 6249 Chrono-environnement, Université de Bourgogne Franche-Comté, Besançon, France; Universitatsklinikum Munster, GERMANY

## Abstract

Antibiotic resistance is a major public health problem requiring the early optimization of antibiotic prescriptions. Matrix-Assisted Laser Desorption Ionization-Time Of Flight Mass Spectrometry (MALDI-TOF MS) has been shown to accurately identify bacteria from positive blood culture. Here, we developed a rapid detection of *Escherichia coli* resistance to amoxicillin (AMX) and cefotaxime (CTX) from positive blood culture based on MALDI-TOF MS. Potential sparing of broad-spectrum antibiotics was also evaluated. We tested 103 *E*. *coli*-positive blood cultures. Blood cultures were subculture 1-hour in antibiotic-free rich liquid media before further incubation with and without AMX for 2.5 h or CTX for 2 h. Protein extracts associated with an internal control were spotted on a MALDI-TOF target and spectra were analyzed with the MBT-ASTRA prototype software (Bruker Daltonik GmbH, Bremen, Germany). Bacterial growth ratio was calculated from the AUC spectra obtained in the presence and absence of the antibiotic and compared to a threshold which classified *E*. *coli* as susceptible or resistant. Results were interpreted with MICs determined using agar dilution method as reference technique. MBT-ASTRA recognized 95% and 84% of the AMX- and CTX-susceptible isolates, respectively. Overall, quantitative analysis of mass spectra allows susceptibility testing within 4 hours after the positivity of blood culture with *E*. *coli*. At the first report of positive blood culture, MALDI-TOF MS would then provide the prescribers with the bacterial identification and the susceptibility to AMX and CTX, thus limiting the use of broad-spectrum compounds.

## Introduction

In recent decades, antibiotic resistance has become a major public health problem requiring the optimization of antibiotic prescriptions. However, the current methods for the determination of bacterial resistance require time. Indeed, after bacterial identification, antibiotic testing needs from 7 h for automated systems with fast-growing species [1] to 24 h for agar dilution method with a return to the clinician the next day [2]. Simultaneously, the increasing proportion of blood infections with antibiotic-resistant *Enterobacteriaceae* limits treatment options [3]. The antibiotic susceptibility testing should be reported as soon as possible to avoid the overuse of broad-spectrum antibiotics (*i*.*e*. third-generation cephalosporins, carbapenems) and to ensure the timely appropriate antibiotherapy. Hence, rapid antibiotic testing is an area of intense research.

Recently, Matrix-Assisted Laser Desorption Ionization-Time Of Flight Mass Spectrometry (MALDI-TOF MS) has become a valuable tool in clinical laboratories for rapid microorganism identification, from colonies or directly from positive blood vials samples [4, 5]. MALDI-TOF MS has also been used for (*i*) the sub-species identification or typing (*ii*) and the detection of particular β-lactamase-based resistance [6–8]. However, most resistances are based either on enzymatic mechanisms which search is not validated by MALDI-TOF MS or on non-enzymatic mechanisms (*e*.*g*. impermeability, target mutations, antibiotic modification, active efflux).

A preliminary study showed that the susceptibility of *Klebsiella pneumoniae* to meropenem can be determined within 1 hour with the new method named MALDI Biotyper-Antibiotic Susceptibility Test Rapid Assay (MBT-ASTRA) [9]. This test is independent from the resistance mechanisms since based on the comparison of bacterial growth with and without an antibiotic.

A recent analysis compared cost-effectiveness of rapid laboratory testing for the diagnosis of bloodstream infections [10]. It found that MALDI-TOF MS species identification associated with antimicrobial stewartship program was the most cost-effective strategy (in terms of reduced mortality probability, length of stay, and cost). From that, we wanted to use MALDI-TOF MS technology not only to identify the pathogen directly from the positive blood cultures but also to test the efficacy of keystone antibiotics, allowing faster adaptation of the treatment. We then adapted the MBT-ASTRA method to the susceptibility testing of *E*. *coli* to amoxicillin and cefotaxime directly from positive blood cultures. Our goal was (*i*) to simultaneously report the bacterial species and the therapeutic options to the clinician (*ii*) to evaluate the potential sparing of broad-spectrum antibiotics.

## Materials and methods

### Bacterial isolates

We carried out the study in 2 steps for both amoxicillin (AMX) and cefotaxime (CTX): (*i*) technique development on blood vials (negative after 5 days of incubation at 35°C) inoculated with clinical *E*. *coli* isolates (set 1), (*ii*) implementation to *E*. *coli*-positive blood cultures of patients (set 2). All the isolates were identified as *E*. *coli* by MALDI-TOF MS Microflex LT with a log score value ≥ 2 according to the manufacturer's recommendations (Bruker Daltonik GmbH, Bremen, Germany). The susceptibility to AMX and CTX was determined by agar dilution method according to the CLSI recommendations [11].

### Technique development on negative blood cultures inoculated with *E*. *coli* isolates

We used 123 non-duplicate clinical isolates of *E*. *coli* collected in the University Hospital of Besançon (France) between 2011 and 2016 and stored at the Centre de Ressources Biologiques Filière Microbiologique, Besançon (CRB-FMB, Biobanque BB-0033-00090). Table 1 details the susceptibility of *E*. *coli* isolates to AMX and CTX. We seeded blood-containing vials (BD BACTEC plus Aerobic/F medium, Becton Dickinson, Sparks, USA) with *E*. *coli* isolates for a final load of 10^6^ CFU. Vials were incubated at 35°C in an automated blood culture system (BACTEC FX Blood Culture System, Becton Dickinson) until its detection.

**Table 1:** *E*. *coli* isolates used to develop the technique on blood-containing vials. The susceptibility to amoxicillin and to cefotaxime was determined by agar dilution method according to the CLSI recommendations [12].

	Amoxicillin		Cefotaxime		
	Susceptible ≤ 8 mg/l	Resistant > 8 mg/l	Susceptible (≤ 1 mg/l)	Intermediate (2 mg/l)	Resistant (> 2 mg/l)
***E*. coli isolates used to spike sterile blood cultures**^a^					
**Clinical isolates**	29	23	30	1	8
**Environmental isolates**	0	9	0	4	19
**Total**	29	32	30	5	27
***E*. *coli* isolates retrieved from blood cultures**^b^					
**Clinical isolates**	40	63	89	3	11

^*a*^ Set 1, isolated between 2011 and 2016.

^*b*^ Set 2, isolated between April and November 2017.

### Implementation to *E*. *coli*-positive blood cultures of patients

We included 103 patients (age > 18 years) hospitalized in the University Hospital of Besançon between April 2017 and November 2017 with a blood culture positive with *E*. *coli* (Table 1).

### Samples preparation for MALDI-TOF MS

During the development and implementation steps, 200 μl of *E*. *coli*-containing blood culture were transferred in a pre-warmed Brain Heart Infusion (BHI) broth and incubated at 37°C for 1 h under agitation (180 rpm). Bacterial suspension was adjusted to 1 McFarland with BHI and further incubated with or without antibiotic (see S1 Fig). We found the optimal antibiotic concentrations and incubation times in preliminary studies: 8 mg/l for 2.5 h for AMX, and 20 mg/l for 2 h for CTX [13]. Blood products were eliminated with steps of centrifugation and washing (see S1 Fig). Bacteria were lysed with formic acid and acetonitrile containing an internal control as previously described [9]. The internal control allows the quantitative comparaison of the spectra with 2 peaks (*m/z* 13,600 and double charged ion *m/z* 6,800). One μl of the *E*. *coli* protein extracts were deposited in duplicate on a MALDI-TOF target. Spots were overlaid with 1 μl of HCCA matrix solution (α-cyano-4-hydroxy-cinnamic acid matrix in 50% acetonitrile with 2.5% trifluoroacetic acid, Bruker Daltonik GmbH) and air dried (see S1 Fig).

### MALDI-TOF MS measurements

We acquired the spectra with a MALDI-TOF MS MicroFlex LT (Bruker Daltonik GmbH) in the linear positive ion mode, with a laser frequency of 60 Hz. Parameter settings for Microflex LT were: ion source 1: 20 kV, ion source 2: 18.5 kV, lens: 6 kV, pulsed ion extraction: 100 ns. The MALDI-TOF analysis used spectra of medium molecular masses ranging from *m/z* 2,000 to 20,000. Each series of analysis was preceded by a calibration with a Bacterial Test Standard (Bruker Daltonik GmbH) that contains an *E*. *coli* reference strain along with RNase A and myoglobin.

### Data analysis

**Fig 1:**
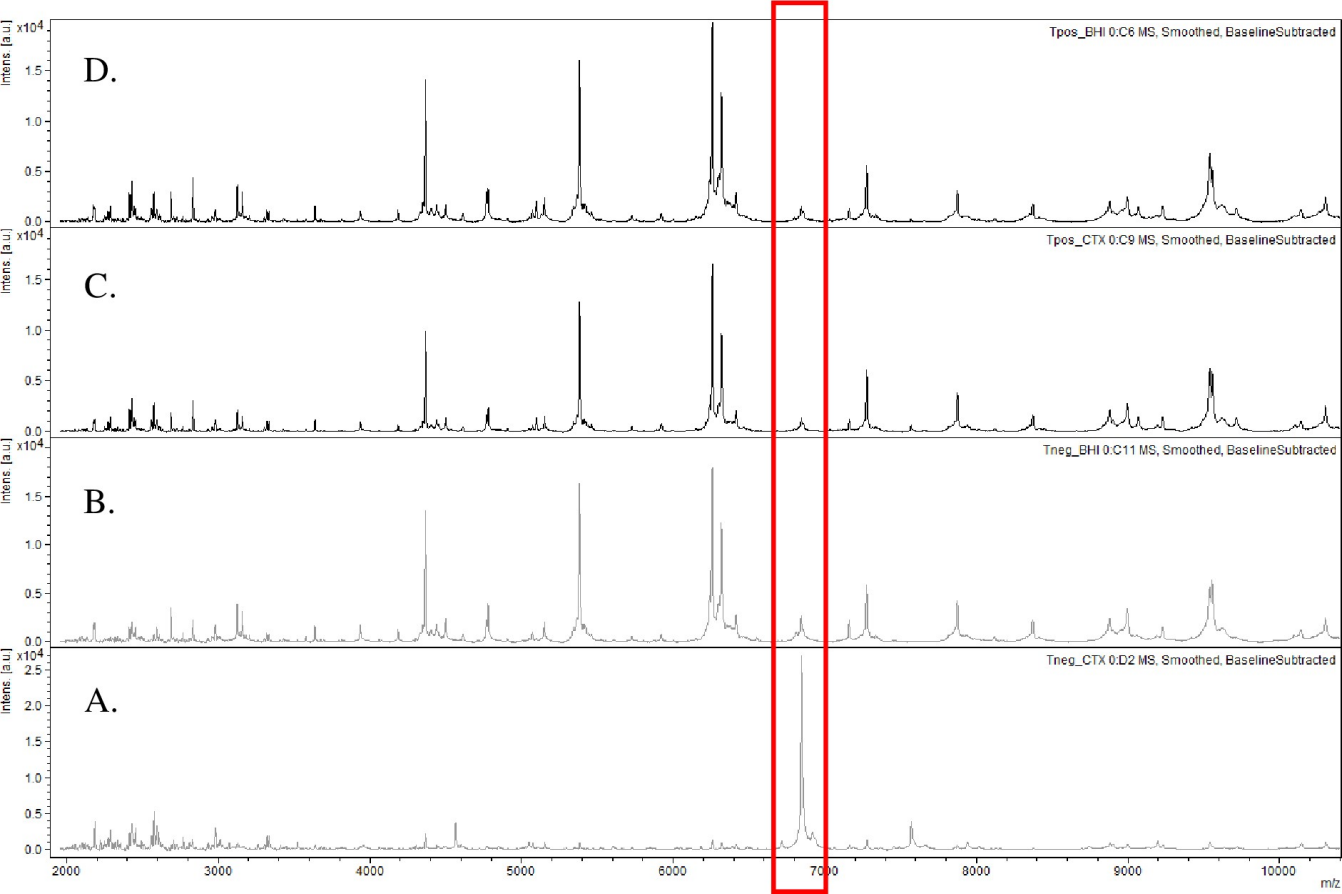
Susceptibility of *E*. *coli* to AMX and CTX revealed by *m/z* 2000–10000 AUC with MS-ASTRA (Bruker Daltonik GmbH). The red rectangle indicate one peak of the internal standard (*m/z* 6,800) used for the quantitative comparaison of the spectra. (A, B) Spectra acquired after 2-h incubation of a CTX-susceptible *E*. *coli* isolate at 37°C with (A) and without (B) 20 mg/l of CTX. (C, D) Spectra acquired after 2-h incubation of a CTX-resistant *E*. *coli* isolate at 37°C with (C) and without (D) 20 mg/l of CTX. The spectra obtained with AMX-S and AMX-R *E*. *coli* incubated 2.5 h at 37°C with 8 mg/l of AMX are stricly superposable.

**Fig 2:**
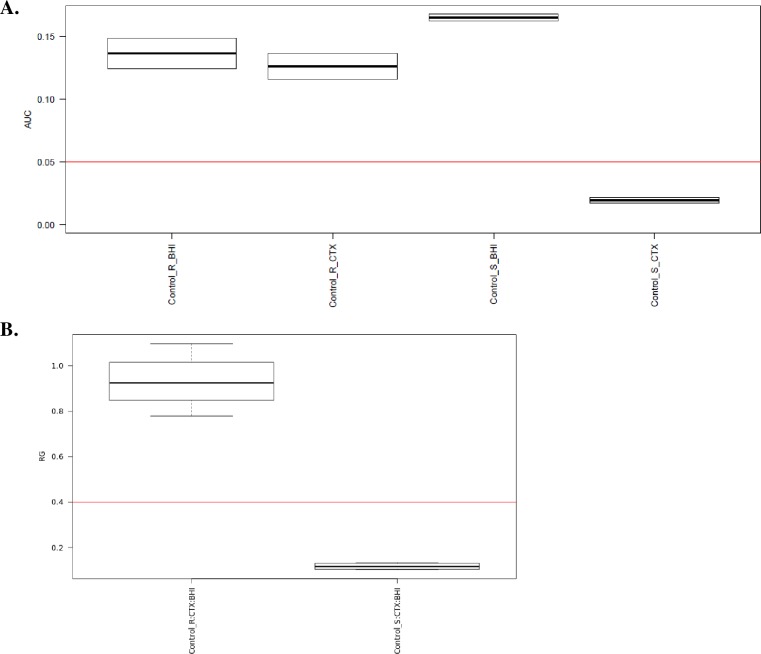
Spectra analysis with MBT-ASTRA prototype software (Bruker Daltonik GmbH). (A) The area under the curve (AUC) of spectra of 2 *E*. *coli* isolates Susceptible (S) and resistant (R) to CTX, with (Control_S_CTX, Control_R_CTX) and without the antibiotic (Control_S_BHI, Control_R_BHI), respectively. The AUC, normalized with an internal control, was calculated automatically by the software through a “calculation window” m/z 2000–10000. (B) A bacterial growth ratio (GR) was automatically calculated by the software from the AUC spectra obtained in the presence and absence of CTX and compared to a threshold which classified *E*. *coli* as susceptible (GR ≤ 0.4 for Control_S.CTX:BHI) or resistant (GR > 0.4 for Control_R.CTX:BHI).

Spectra were analyzed with the MBT-ASTRA prototype software (Bruker Daltonik GmbH) which estimates the bacterial growth. The incubation of an isolate with an active antibiotic reduces the intensity and the number of the peaks detected by MALDI-TOF MS. On the other hand, the incubation of an isolate with an inactive antibiotic will not modify the spectra (Fig 1). The area under the curve (AUC) of samples spectra with and without antibiotics was automatically calculated by the software through a “calculation window” *m/z* 2000–10000. Thus, when normalized with a remaining internal control, the antibiotic-susceptible isolate AUC is reduced when incubated in the presence of this antibiotic (Fig 2A). For each assay, we calculated a growth ratio (GR) from the AUC spectra obtained in the presence and absence of antibiotic and compared to a threshold (Fig 2B). Preliminary studies identified a relative growth rate of 0.4 as a threshold that distinguishes susceptible from resistant isolates [9, 13]. The MBT-ASTRA results were compared to the MIC values measured with the reference method. Indicators of the performance of the diagnostic tool (sensitivity, specificity) were calculated with Open Epi software version 3.01 (www.openepi.com).

### Potential sparing of broad-spectrum antibiotics

We compared antibiotic treatments received by the patients (first 48 h) with treatments that could have been prescribed if the resistance to AMX and CTX had been assessed directly from the positive blood vials. We collected retrospectively the data from medical records and from the pharmacy software. For each patient with a *E*. *coli-*positive blood culture over the duration of the study (April to November 2017), we recorded demographic and clinical data, concomitant infectious diseases, prescribed probabilistic antibiotic treatment, prescribed antibiotic treatment after blood culture sampling and after information of the clinician of bacterial identification and resistance. These data were analyzed with regards to the MICs and MBT-ASTRA results. Since the present technique was under evaluation, the results had not been transmitted to the clinicians during this study.

### Ethics statement

The study was approved by the ethical committee ‘Comité d’Etude Clinique’ of the Besançon hospital, Besançon, France.

## Results

### Sterile blood vials inoculated with *E*. *coli*

We evaluated the performance of MBT-ASTRA technique for the rapid AMX and CTX susceptibility testing. Using MIC determination as a gold standard, for the detection of resistance to AMX we found that 97% of the isolates (59 out of 61) were correctly classified by the MBT-ASTRA technique with a sensitivity of 100% and a specificity of 93% (Table 2). Two AMX-susceptible isolates (with MICs of 4 and 8 mg/l) were classified as AMX-resistant by MBT-ASTRA. For the detection of resistance to CTX, MBT-ASTRA correctly classified 94% of the isolates (58 out of 62) with sensitivity and specificity of 94% and 93%, respectively. Two CTX-susceptible isolates (with MICs of 0.25 and 1 mg/l) were classified as resistant by our technique. Another two CTX-resistant isolates (with MICs of 2 and 8 mg/l) were found susceptible by MBT-ASTRA. For both AMX and CTX susceptibility testing the full agreement was to 93% with a very major error of 0% and 6%, respectively (Table 3).

**Table 2:** Performance of MBT-ASTRA (Bruker Daltonik GmbH) for AMX and CTX susceptibility testing of *E*. *coli*-positive blood cultures.

	**MBT ASTRA**	
	**AMX-susceptible** ***n***	**AMX-resistant** ***n***
**Sterile blood cultures inoculated with *E*. *coli***		
**AMX-susceptible**	27	2
**AMX-resistant**	0	32
***E*. *coli* isolates retrieved from blood cultures**		
**AMX-susceptible**	37	2
**AMX-resistant**	1	63
	**CTX-susceptible** ***n***	**CTX-resistant** ***n***
**Sterile blood cultures inoculated with *E*. *coli***		
**CTX-susceptible**	28	2
**CTX-resistant**	2	30
***E*. *coli* isolates retrieved from blood cultures**		
**CTX-susceptible**	75	14
**CTX-resistant**	4	10

**Table 3:** Categorical agreement of MBT-ASTRA (Bruker Daltonik GmbH) for AMX and CTX susceptibility testing of *E*. *coli*-positive blood cultures.

	Sterile blood cultures inoculated with *E*. *coli*		*E*. *coli* isolates retrieved from blood cultures	
	AMX test	CTX test	AMX test	CTX test
**Full agreement**	93%	93%	95%	84%
**Minor error**	Non applicable	Non applicable	Non applicable	Non applicable
**Major error**	7%	7%	5%	16%
**Very major error**	0%	6%	2%	29%

### Positive blood cultures with *E*. *coli*

The overall categorical agreement between MBT ASTRA method and the reference method was 97% (100 isolates out of 103) and 83% (85 isolates out of 103) for AMX and CTX, respectively (Table 2). The MBT ASTRA identified AMX- and CTX-susceptible isolates with a sensitivity of 98% and 71%, respectively. One AMX-resistant isolate (MIC, 12 mg/l) and four CTX-resistant isolate (MICs of 1, 2, 4, and 32 mg/l) were found susceptible by MBT-ASTRA. The full agreement was 95% and 84% with a very major error of 2% and 29%, for AMX and CTX, respectively respectively (Table 3).

### Potential sparing of broad-spectrum antibiotics

Among 89 infections with a CTX-susceptible *E*. *coli*, de-escalation of antibiotic treatments was achieved for 16 patients. No adaptation was done for 39 patients. In 34 cases, either data on the antibiotic treatment were missing from medical charts or antibiotic sparing was impossible due to associated infections. Of the 16 cases where antibiotic de-escalation was performed, 11 isolates were AMX-susceptible, and 5 isolates were AMX-resistant. An early report with MBT-ASTRA would have saved 8 and 7 days of third-generation cephalosporins and piperacillin-tazobactam respectively. Among the 39 patients who had not been de-escalated, MBT-ASTRA would have saved 112, 40, and 12 days of third-generation cephalosporins, piperacillin-tazobactam, and carbapenems, respectively.

## Discussion

MBT ASTRA method indirectly estimates the growth of bacterial strains. The peak normalization combined with an internal quantitative control permits the comparison of the total intensity of the MALDI-TOF MS spectra. The peak intensity reflects the amount of bacterial proteins, which directly depends on the bacterial growth [9]. Recently MBT-ASTRA assay was implemented in several studies for the detection of bacterial resistance either from clinical isolates or directly from positive blood cultures [9, 13–15]. Gram-negative bacterial species (*Enterobacteriaceae*, *Pseudomonas sp*., and *Acinetobacter sp*.) were tested in combination with different antibiotic classes (penicillins, cephalosporins, carbapenems, fluoroquinolones, or aminoglycosides) [13, 14]. *Mycobacteria tuberculosis* was tested with rifampicin and izoniazid, respectively [15]. The protocol optimization (*i*.*e*. antibiotic concentration and incubation time) is necessary for each species-antibiotic combination. Unfortunately, the antibiotic (ATB) concentrations to be tested (ATB_MS-ASTRA_) cannot be predicted from the susceptible clinical breakpoints (ATB_S breakpoint_). For instance, the ratio CTX_MS-ASTRA_/CTX_S breakpoint_ varies from 2 to 20 depending on the bacterial species and studies [13, 14]. Moreover, the optimal incubation time depends on the nature and the concentration of the antibiotic and varies from 1 to 4 h.

We present here a procedure for the rapid susceptibility testing of *E*. *coli* to AMX and CTX directly from positive blood culture vials. Early information of the susceptibility to these compounds is necessary to spare broad-spectrum antibiotics such as third-generation cephalosporins, piperacillin-tazobactam, and carbapenems. Despite many attempts, we failed to find optimal testing conditions for testing susceptibility to piperacillin-tazobactam with MBT ASTRA, possibly due to an initial plateau phase in the time-kill curves of piperacillin or to the instability of the antibiotic association [13, 14].

MBT ASTRA method was first optimized using a set of sterile blood vials inoculated with *E*. *coli* (set 1) and further implemented on *E*. *coli*-positive blood cultures (set 2). The MBT- ASTRA assay testing AMX was highly sensitivity (100% and 98%) and reasonably specific (93% and 95%) with set 1 and 2, respectively. This contrasted with the assay testing CTX. Hence, while the sensibility and the specificity with set 1 was good (94% and 93%, respectively), the sensibility and the specificity was poor using *E*. *coli*-positive blood cultures (71% and 84%, respectively). The discrepancy between the sets is presumably due to the low proportion of CTX-resistant isolates in set 2. The correlation between the relative growth value and the MIC of CTX could be ameliorated with the extension of the incubation time but makes the test less attractive.

Rapid antibiotic susceptibility testing is an area of intense interest [16–18]. Here, we found that MBT-ASTRA test can accurately (*i*) determine the susceptibility of *E*. *coli* to AMX and (*ii*) identify CTX-susceptible *E*. *coli* directly from positive blood culture in less than 4 h, independently from the resistance mechanism. In the same workflow, species identification with MALDI-TOF MS and rapid antibiotic susceptibility testing with MBT-ASTRA can significantly reduce the time to re-evaluate the antibiotic therapy. Our data confirm that MBT-ASTRA approach is promising [13–15]. MALDI-TOF MS technology is already well established in clinical laboratories and the MBT-ASTRA test is within the capabilities of the laboratory technicians. The development of commercial and automated kits with keystone antibiotics would be useful to quickly orientate the treatment of high-risk patients.

We also assessed the potential sparing of broad-spectrum antibiotics. The most important point to emphasize is that the MBT ASTRA concept would streamline the information transmitted to prescribers. Shortly after the detection of positive vials by automates, they would be informed of the pathogen identification together with its susceptibility to keystone antibiotics, which favors the prescription of the active antibiotic with the narrowest spectrum. However, the potential sparing of broad-spectrum antibiotics using MBT ASTRA remained to be demonstrated in real life and depends on the commitment of prescribers in antimicrobial stewardship policies.

## Supporting information

S1 Fig Sample preparation for MALDI-TOF MS detection of antibiotic resistance with MBT-ASTRA method. 200 μl of E. coli-positive blood cultures were transferred in a pre-warmed BHI broth and incubated at 37°C for 1 hour. The density of the bacterial culture was adjusted to 1 McFarland with BHI and further incubated at 37°C with and without the antibiotic to test. After washing, formic acid, acetonitrile and internal control were added. The supernatant was spotted on a MALDI-TOF target and overlaid with HCCA (Bruker Daltonik GmbH). Bacterial growth ratio was calculated from the AUC spectra obtained in the presence and absence of the antibiotic and compared to a threshold which classified E. coli as susceptible or resistant.
(DOC)
