# Supplementary material for: Rapid antibiotic susceptibility testing on blood cultures using MALDI-TOF MS
Source: PLoS One. 2018 Oct 11;13(10):e0205603. doi: 10.1371/journal.pone.0205603 (PMC6181389; doi:10.1371/journal.pone.0205603)
Supplement: S1 Fig — 200 μl of E. coli-positive blood cultures were transferred in a pre-warmed BHI broth and incubated at 37°C for 1 hour. The density of the bacterial culture was adjusted to 1 McFarland with BHI and further incubated at 37°C with and without the antibiotic to test. After washing, formic acid, acetonitrile and internal control were added. The supernatant was spotted on a MALDI-TOF target and overlaid with HCCA (Bruker Daltonik GmbH). Bacterial growth ratio was calculated from the AUC spectra obtained in the presence and absence of the antibiotic and compared to a threshold which classified E. coli as susceptible or resistant. (DOC) [file pone.0205603.s001.doc]

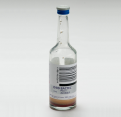


**Adjust to 1 McF in BHI**

**200 μl in warm BHI**

**Incubation**

**(900 rpm, 37 °C**

**2 h for CTX or**

**2.5 h for AMX)**

**100 μl**

**Centrifugation**

**(18,000 X *g* for 2 min)**

**+**

**Cleaning with 100 μl of pure water**

**Stirring**

**(180 rpm, 37 °C, 1 h)**


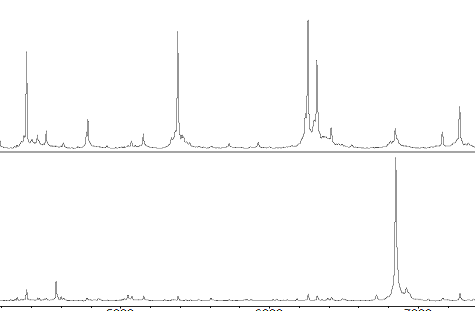


**Add 100 μl of BHI**

**Add 100 μl of antibiotic :**

**CTX final concentration 20 mg/l**

**Or AMX final concentration 8 mg/l**


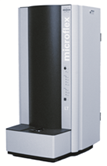


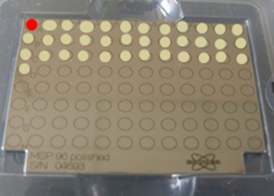


**Centrifugation**

**(18,000 X *g* for 2 min)**

**+ 10 μl formic acid**

**+ 10 μl acetonitrile with internal control**

**Centrifugation**

**(18,000 X *g***

**for 2 min)**

**Supernatant**

**+ HCCA spot**

**Spectrum**

**acquisition**

**Spectrum analysis**
